# On the Use of Nanoparticles in Dental Implants

**DOI:** 10.3390/ma17133191

**Published:** 2024-06-29

**Authors:** Liliane Bokobza

**Affiliations:** Independent Researcher, 194-196 Boulevard Bineau, 92200 Neuilly-sur-Seine, France; liliane.bokobza@wanadoo.fr

**Keywords:** dental implants, titanium, nanoparticles, nanocoatings, sol–gel process, titanium dioxide, carbon nanotubes, graphene-based materials, toxicity

## Abstract

Results obtained in physics, chemistry and materials science on nanoparticles have drawn significant interest in the use of nanostructures on dental implants. The main focus concerns nanoscale surface modifications of titanium-based dental implants in order to increase the surface roughness and provide a better bone–implant interfacial area. Surface coatings via the sol–gel process ensure the deposition of a homogeneous layer of nanoparticles or mixtures of nanoparticles on the titanium substrate. Nanotubular structures created on the titanium surface by anodic oxidation yield an interesting nanotopography for drug release. Carbon-based nanomaterials hold great promise in the field of dentistry on account of their outstanding mechanical properties and their structural characteristics. Carbon nanomaterials that include carbon nanotubes, graphene and its derivatives (graphene oxide and graphene quantum dots) can be used as coatings of the implant surface. Their antibacterial properties as well as their ability to be functionalized with adequate chemical groups make them particularly useful for improving biocompatibility and promoting osseointegration. Nevertheless, an evaluation of their possible toxicity is required before being exploited in clinical trials.

## 1. Introduction

The advent of nanoparticles with sizes between 1 and 100 nm, at least in one dimension, has generated huge interest in the last few decades on account of their wide range of potential applications in many areas, including materials, medical and dental sciences. One typical application in the field of materials science is rubber nanocomposites that exhibit significantly improved mechanical, electrical and thermal properties with regard to the pristine compound, thus contributing to the development of modern tire technology [1,2,3,4]. Nanosized particles have gained significant importance in medicine for their ability to deliver drugs to specific cells, which allows for therapy of several diseases [5,6]. Important progress has been achieved in the field of oncology by targeting the cancer cells, thus reducing the toxicity of the conventional chemotherapy treatments and the damage of healthy cells. Moreover, functionalization of the nanoparticles by grafting appropriate chemical groups on their surface allows for the synthesis of innovative nanocarriers with better targeting efficiency [7].

The use of nanoparticles in dentistry, especially in the field of dental implants, opens the way to new strategies for a better integration of the implants into the bone and for solving side effects occurring after implantation [8,9,10,11,12,13,14,15].

Titanium has been shown to be the ideal material for dental implants on account of its mechanical properties, biocompatibility and great corrosion resistance as well as its bonding to the osseous tissue, known as osseointegration [16,17,18]. The process of osseointegration depends on several factors, namely the implant characteristics, such as the shape, length, diameter and, essentially, the surface properties that determine the bone–implant interface. A higher bone–implant interface has been observed with rough implant surfaces [19]. It has also been demonstrated that titanium is covered by a titanium dioxide layer arising from the contact between titanium and oxygen [20]. This oxidized layer that bridges the metal to the living bone imparts corrosion resistance and the stability of the connection between the two phases [21].

The quality of bone surroundings also affects implant success and, unfortunately, frequent failures such as inflammation and infection may occur as a result of a poor bone–implant adhesion that leads to bacterial infiltration. Various studies aiming at reducing these implant failures tried to increase the surface roughness of the implant by performing nano-topographic modifications in order to obtain nanostructures on the titanium surface. The nanoscale roughness can be achieved by the use of nanoparticles that can improve the osseointegration process through a higher interfacial area [10,22,23]. In addition to the use of nanoparticles, as only considered in this study, it has to be mentioned that dental implants can also be treated by polymer coatings. Several biodegradable polymers and especially chitosan have been shown to provide biocompatibility, non-toxicity, antimicrobial and anti-corrosion properties as well as osseointegration capability [24,25,26].

In this paper, the recent advances in nanoscale surface modifications are reviewed. We will concentrate on nanocoatings formed by titanium dioxide, hydroxyapatite and carbon-based materials (carbon nanotubes, graphene and its derivatives), but the use of other metal oxide nanoparticles in dental applications are mentioned in several studies [9,10,12,27,28].

## 2. Micro-Scale Modifications of Dental Implant Surfaces

Of primary interest is the implant design that determines the mechanical stability, which is essential for successful osseointegration. Implant design involves the type of material, the neck and the apex geometry, the body characteristics (length, diameter and shape), the thread pattern, the implant–abutment connection and the surface treatment. The design evolution of dental implants aims to maximize the bone–implant contact for a better anchorage by decreasing, for example, the thread pitch or by increasing the surface roughness.

Dental implants are mostly manufactured in pure titanium or in titanium alloys. Machined surfaces appear macroscopically smooth but display microgrooves and microrugosities along the machining direction. But commercial dental implants are available with many different surface topographies obtained by mechanical, chemical, plasma or laser treatments [29].

Grit blasting is one of the most used mechanical techniques to create textured surfaces due to the impact of hard particles such as alumina, silica or titania. These particles in a size range 25–250 μm yield a surface roughness between 1 and 2 μm.

Chemical etching uses generally strong acids, such as nitric, sulfuric, hydrochloric, or hydrofluoric acids, that create dips to allow for bone growth. The etching process has been shown to create a micro-roughness between 0.5 and 2 μm and to depend on the acid concentration, temperature and etching time [30]. An optimal roughness is often obtained by combining grit blasting and acid etching [31,32]. Moreover, surface implants being treated by blasting followed by double acid etching lead to a dual roughness, favorable for a better osseointegration [31,33,34].

Plasma treatments that use partially ionized gases are divided into thermal and non-thermal (cold atmospheric plasma) plasmas [35,36]. The thermal plasma technology has been applied to deposit a coating material such as hydroxyapatite on the titanium implant surface (plasma spraying), resulting in the formation of a layer 50–100 μm thick [37,38]. Cold atmospheric plasma can be generated by various devices, including dielectric barrier discharges at atmospheric pressure, plasma pencils, plasma needles, plasma brushes and plasma jets [39,40,41]. This technique has several applications in dentistry, such as sterilization of dental instruments, disinfection of dental cavities, tooth bleaching, biofilm removal and implant surface modification. Regarding implantology, plasma treatment has been shown to increase the surface energy, which improves roughness and wettability in addition to its antimicrobial activity [42].

Laser ablation—a process for the removal of a material from a solid—has emerged as an innovative implant manufacturing technology for its ability to create clean and precise micro-scale patterns, perfectly reproducible by controlling the processing laser parameters. Laser surface texturing is obtained by the irradiation of the implant area conducted by a focused laser beam operating at a given wavelength and directed normal to the target surface. This orthogonal configuration between the laser beam and the surface has been shown to have a maximized effect in reducing biofilm formation [43]. Additionally, as well as reducing contamination, laser ablation enhances osseointegration and improves connective tissue attachment [44,45].

## 3. Nanoscale Modifications and Coatings of Dental Implant Surfaces by Nanoparticles

As already mentioned, an improvement in the surface properties of titanium dental implants can be achieved by the use of nanoparticles on account of their nanoscale dimension enabling the creation of a large interfacial area on the substrate material for a better osseointegration. The performance of coatings on the implant surface by nanoparticles is expected to depend on the nanoparticle characteristics, including the particle size, structure and surface chemistry. Different nanoparticles including isotropic (nanospheres) or anisotropic with a one- or two-dimensional nature such as sheet-like (obtained from exfoliation of layered structures) and rod-shaped (nanotubes) morphologies will be considered here.

### 3.1. Titanium Dioxide (TiO_2_) Nanocoatings

TiO_2_ nanocoatings have been widely used to improve the corrosion resistance of the titanium implants, and their nanostructures highly depend on the synthesis routes.

The sol–gel process is a simple and efficient method to generate small and non-agglomerated particles and perform homogeneous coatings on the implant surface. It involves inorganic alkoxides ([M (OR)_x_] called precursors, where M = Si, Ti, Zr, Al,. … and R is an aliphatic group, which are all hydrolyzed and condensed under acidic or basic conditions, in order to form M-O-M bridging units. As an example, titanium isopropoxide [46,47] or titanium n-butoxide [48] has been used as precursors of titanium dioxide, TiO_2,_ in the sol–gel process. The structure of the synthesized particles depends on the hydrolysis and condensation conditions and also on the pH of the medium. Films of titanium dioxide can be homogeneously deposited on the substrate by dip coating or spin coating, and the thickness can be increased by repeated treatments [49,50,51,52].

**Figure 1:**
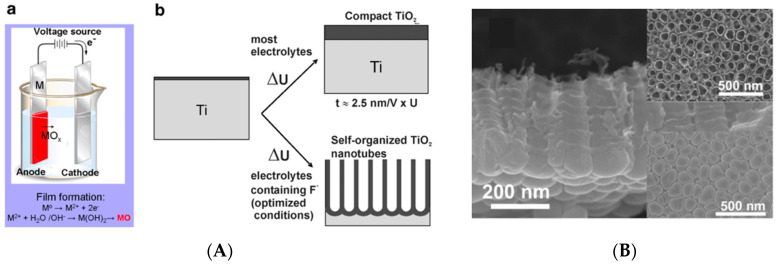
(**A**): Experimental set-up for electrochemical anodization of titanium substrate for the formation of a compact oxide layer (a) or for nanotubes (b). (**B**): SEM image of TiO_2_ nanotubes grown in fluoride containing electrolyte. Source: Reprinted with permission from Ref. [54].

The research of new nanomaterials for successful osseointegration leads to the use of nanostructures with different morphologies, able to impart new properties and new functionalities. Of special interest are nanotubular particles of TiO_2_ on account of their ordered structure, their high surface area and roughness and their ability to be used as drug carriers. Anodic oxidation of titanium and its alloys is well known to yield a compact oxide layer or highly ordered porous structures depending on the electrolyte composition and on the anodization conditions [52,53,54,55,56,57,58,59,60,61]. Typically, electrochemical anodization uses a dual-electrode system (the Ti implant serves as the anode and a Ti/platinum as the cathode of an electric circuit) immersed in an electrolyte. The solid TiO_2_ layer grown on the metal surface by anodization is thicker (some hundred nanometer thick), roughened and less defective than the thin layer (a few nanometers thick) resulting from the spontaneous oxidation of titanium in air. In the presence of fluoride ions in the electrolyte, self-organized nanotubular or nanoporous structures are formed on the titanium surface, as shown in Figure 1. These nanoporous structures are strongly affected by the anodization parameters (applied potential, electrolyte composition, temperature, pH).

The mechanism of formation for nanotubular structures by electrochemical anodization has drawn considerable attention. It is reported that the compact TiO_2_ layer is formed by the reaction of Ti^4+^ (ejected by the applied electric field from the Ti substrate to the electrolyte) with the O^2−^ ions from the dissociation of H_2_O. Ti^4+^ can also react with the fluoride ions to form [TiF_6_]^2−^ complexes able to dissolve the compact TiO_2_ layer, giving rise to the porous and tubular structures [54,55,60,61]. Regonini et al. [55] point out the role played by the nature of the electrolyte (organic, aqueous, water content in organic solutions) on the morphology of the nanotubes. It has been shown that fluoride ions in water-based solutions yield nanotubes with ripples and lengths limited to a few microns, while those grown in organic electrolytes containing fluoride salts and small amount of water are highly self-organized, with no ribs on the tube sides and lengths up to 100–1000 μm.

**Figure 2:**
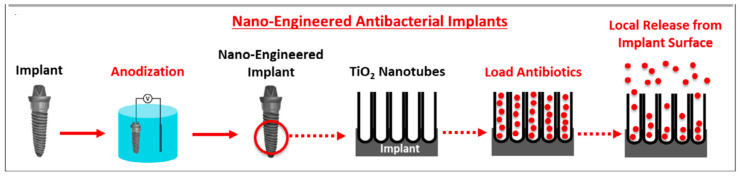
Schematic representation of therapeutic application of titania nanotubes in dental implantology. Source: Reprinted with permission from Ref. [65].

These nanotubular structures created on the titanium surface have been the subject of several tests in order to evaluate their therapeutic efficiency. Ercan et al. [62] found that the nanosurface modification of titanium provides antibacterial effects. Investigations carried out on nanotubes of highly controlled diameters (obtained through a change in the anodization parameters) and submitted or not to a heat treatment led the authors to conclude that the heat-treated and 80 nm diameter titanium tubes produced the most robust antimicrobial effect. The antibacterial functions of the nanotubes are mainly attributed to their ability to be loaded and to release various antibacterial agents and antibiotics (Figure 2). This therapeutic potential and the technical challenges necessary to bridge the gap between research and clinical activity have been widely discussed in the literature [63,64,65]. Metal-doped titania nanotubes have also been shown to exhibit antibacterial effects [64]. Jia et al. [66] applied a thin film of polydopamine to anodized titanium, onto which Ag^+^ ions could be immobilized via chelation to the catechol groups of the polydopamine. The resulting coating, in addition to protecting the substrate from corrosion, allowed for the local release of silver ions up to 28 days and displayed “trap-killing” antimicrobial activity. The release of bactericidal Ag^+^ paves the way for the development of silver-coated dental implants, particularly those exposed to a bacterial biofilm. The antimicrobial properties of silver have been known for many years, and silver ions or silver nanoparticles have been previously used in several medical applications on account of their broad antibacterial spectrum against a wide range of microorganisms [67,68,69]. Recent studies by Rodríguez-Hernández et al. [70] suggest the possible use of magnesium oxide nanoparticles in dental applications due to the antibacterial properties in all oral biofilm strains.

**Figure 3:**
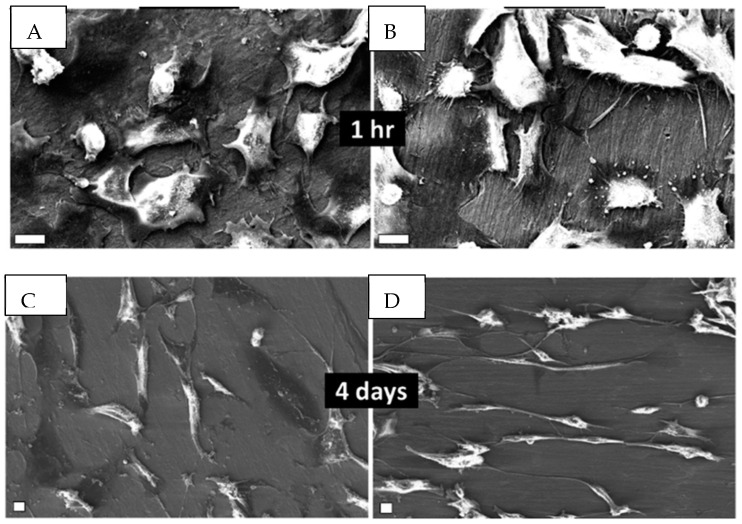
Scan images comparing the time dependence of the adhesion of human gingival fibroblasts in rough Ti (**A**,**C**) and in TiO_2_ nanopores, diameter 70 nm (**B**,**D**). Scale bar: 10 μm. Source: Reprinted with permission from Ref. [71].

An interesting approach is that of Gulati and coworkers [71], which generates a titania nanostructure on a micro-rough titanium surface. Primary macrophages, osteoblasts and fibroblasts were cultured on the nano-engineered surface of the implant. It is demonstrated that this dual micro-nano architecture induced an alignment of fibroblasts and osteoblasts on the nanostructured surface, which is favorable for cell adhesion (Figure 3).

### 3.2. Hydroxyapatite (HA) Nanocoatings

As a major component of bone, hydroxyapatite (HA) has received much attention as a surface coating for titanium, but failed interfacial adhesion between HA and the implant has been observed [72,73]. Renewed interest has been focused on HA again on account of the advent of nanocoatings and on the use of hydroxyapatite-based nanocomposites [74]. Among the various deposition techniques of HA, the sol–gel coating is expected to provide a better layer homogeneity and can be performed at low processing temperatures [75,76,77,78,79,80,81,82,83,84]. But, despite the recent developments in the deposition of HA on titanium implants by the sol–gel process, some limitations of this technique are mentioned in the review of Jaafar et al. [82]. In particular, optimizing the sol–gel parameters (nature of the precursor, pH value, sintering temperature…) is essential for a controlled morphology of the particles and for improving their coating properties. However, weak bonding between the coating and the substrate leading to decohesion may result from a difference in the coefficient of thermal expansion between the two phases [85,86].

**Figure 4:**
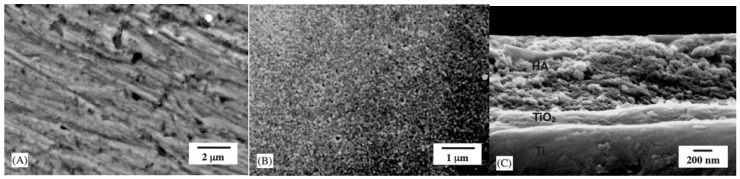
Scanning electron micrographs of different coatings deposited on titanium. (**A**) TiO_2_; (**B**) HA/TiO_2_ double layer; (**C**) cross-section of the HA/TiO_2_ double layer after treatment performed at 500 °C. Source: Reprinted with permission from Ref. [85].

Kim et al. [85] inserted a TiO_2_ layer between HA and the titanium substrate, expected, due to its chemical similarity to both the titanium and hydroxyapatite, to improve the bonding strength of the HA coating. Both layers were deposited on titanium by using a sol–gel approach, and scanning electron microscopy (SEM) revealed different morphologies for the two different coatings and a tight bonding of the inserted titania layer to both Ti and to HA (Figure 4). Moreover, the double-layer coating after heat treatment at 500 °C displays a 60% increase with regard to the single HA coating. Kim et al. [86] also use the sol–gel approach for the synthesis of hydroxyapatite and titania composite coatings on titanium. The composite films coated on a titanium substrate are prepared by using sol–gel precursors of both HA and TiO_2_, and the sols are mixed together at various TiO_2_ ratios up to 30 mol%. The composite coating layers display a homogeneous structure with a roughness and an adhesion strength that increase with the TiO_2_ content.

**Figure 5:**
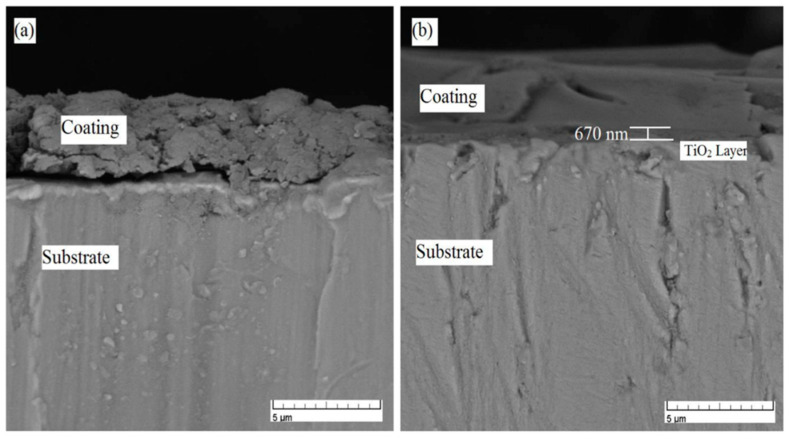
Cross-sectional SEM images of (**a**) HA and (**b**) functionally graded HA-TiO_2_ coatings. Source: Reprinted with permission from Ref. [87].

Following the promising results obtained by Kim et al. [85], with the use of a TiO_2_ middle layer, Azari et al. [87] prepared a functionally graded HA-TiO_2_ coating (100% TiO_2_, 50% TiO_2_-50% HA, 100% HA) by the sol–gel process and a spinning method on a Ti-6Al-4V alloy substrate. The composite layer has upper (HA) and lower (TiO_2_) components in order to create better compatibility with the different phases. The cross-sectional SEM image of the functionally graded coating shows that the insertion of the TiO_2_ layer improves the adhesion with regard to a single-layer HA coating (Figure 5).

Nanocomposite coatings based on HA and nanoparticles of alumina, zirconia or silica .... have also been used to improve mechanical properties and adhesion between the coating and the implant, which has a significant impact on the osseointegration [74,88].

### 3.3. Carbon Nanotube (CNT) Nanocoatings

**Figure 6:**
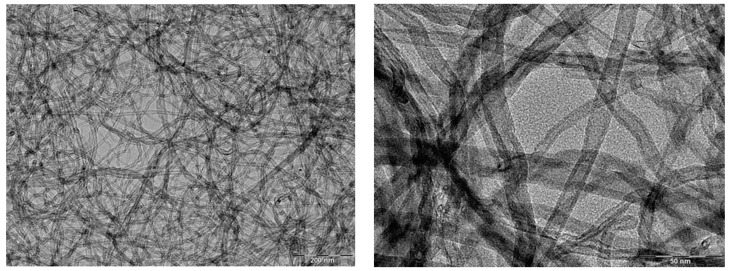
TEM images of a suspension of multiwall carbon nanotubes (MWCNTs) in isopropyl alcohol at two different magnifications (from Bokobza’s own research).

Since their discovery in 1991 by Iijima [89], carbon nanotubes (CNTs) have attracted tremendous attention on account of their outstanding mechanical, electrical and thermal properties that make them ideal nanomaterials in electronic devices and polymer nanocomposites. They can be visualized as graphene layers (a single sheet of sp^2^ carbon atoms packed into a two-dimensional honeycomb lattice) rolled into individual (single-wall carbon nanotubes SWCNTs) or concentric (multiwall carbon nanotubes MWCNTs) cylinders, with typical diameters ranging from about one to tens of nanometers and lengths of several micrometers (even millimeters or centimeters). Figure 6 shows typical transmission electron microscopy (TEM) images of an isopropyl alcohol suspension of MWCNTs previously sonicated before being put onto copper grids for observation. The tubes are highly entangled, which reveals their exceptional flexibility. These carbon-based materials are used in materials science as a reinforcing agent. They have been shown to impart to polymeric systems substantial improvements in stiffness at low filler loadings, but their tendency to form agglomerates as well as the lack of interfacial adhesion between the tubes and the polymer matrix limit the full realization of their superb potential [90,91]. Modification of their surface chemistry through functionalization is often required to improve their state of dispersion in the host medium and to promote a strong polymer–filler interface [92,93]. In addition to their reinforcing capability, carbon nanotubes, as other black fillers, impart electrical conductivity to insulating matrices. But the high aspect ratio (length/diameter) of the tubes allows for the formation of a percolated filler network with a very small carbon nanotube amount (less than 1 wt%), while 10–50 wt% is required in the case of conventional carbon black particles, for example, in rubber matrices [94].

Due to their fascinating properties, carbon nanotubes offer new promise in the field of dental and orthopedic implants as reinforcing agents for implant coatings, drug delivery systems or bone regeneration and repair [95,96,97,98]. The reinforcing capability of carbon nanotubes for implant coatings, especially for brittle hydroxyapatite (HA), has been demonstrated in the literature [99,100,101,102]. Balani et al. [99] used plasma spraying for the synthesis of HA-CNT coatings onto a Ti-6Al-4V implant. The CNTs are uniformly distributed in the HA-4 wt% CNT coating and impart to the coating an improvement in fracture toughness and an increase in crystallinity. The work of Facca et al. [100] reports in vivo investigations of HA-4 wt% CNT-coated Ti-6Al-4V implants in rodents’ bone. No infection, rejection or inflammation reactions were reported, and, contrary to what has often been claimed, no cytotoxicity upon CNT addition on bone tissues and cells was observed. Additionally, a decreasing gradient in the elastic modulus is revealed for both HA and HA-CNT, attributed to the tissue in-growth at the coating–bone interface. In the study of Abrishamchian et al. [101], HA/MWCNT nanocomposite coatings were prepared by a sol–gel method on Ti alloy substrates. Above 1 wt% nanotube content, the surface roughness of the coatings determined by atomic force microscopy (AFM) increased, while a decrease in hardness and elastic modulus was observed, most probably due to a non-uniform dispersion of MWCNTs. This tends to show that unfunctionalized carbon nanotubes have to be used at very low contents to avoid their agglomeration into bundles. The effect of MWCNT functionalization is highlighted in Gholami et al. [102], dealing with HA composites, incorporated with different types of MWCNTs including hydroxylated and carboxylated MWCNTs. Functionalized nanotubes lead to more homogeneous structures and less agglomerated particles, and the highest compressive strength is obtained for the composite prepared with MWCNTs-COOH. Carboxylated MWCNTs were also used by Terada et al. [103] to coat a titanium plate previously aminated and covered with collagen. A significant increase in the surface roughness was observed with the MWCNT coating that homogeneously covers the collagen-coated Ti plate surface without aggregation. Moreover, the MWCNT-coated Ti plate is shown to display stronger cell adhesion than untreated titanium. In the work of Park et al. [104], multiple surface treatments were carried out in order to enhance the bioactivity of pure titanium. Titanium is first treated by anodization that forms TiO_2_ nanotubes on the surface then submitted to an alkali treatment and subsequently coated with carboxylated MWCNTs. The improvement in biocompatibility through the multiple surface treatments is evaluated through various cytotoxicity tests using mouse osteoblast cells.

### 3.4. Nanocoatings of Graphene-Based Materials

Graphene, a single layer of sp^2^ hybridized carbon atoms, considered as the building unit of all graphitic carbon allotropes, has attracted unprecedented interest due, as for carbon nanotubes, to its impressive mechanical and electronic properties. In addition, the single-layer morphology that offers a large surface area, able to achieve more contact with the surrounding medium, makes graphene and its derivatives (graphene oxide, reduced graphene oxide and graphene quantum dots) ideal candidates as fillers for the design of advanced composite materials. However, the state of graphene dispersion in the host medium is of crucial importance to take advantage of the unique properties of the individual sheets.

**Figure 7:**
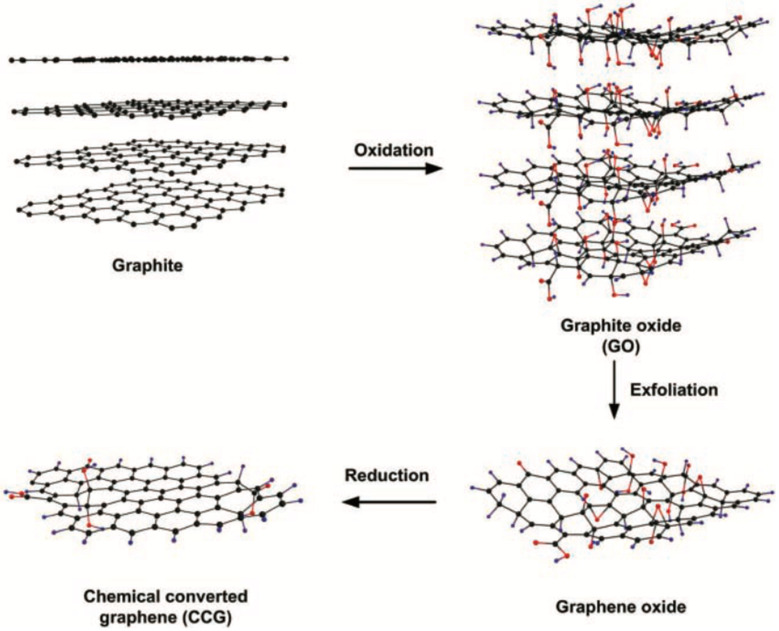
Schematic illustration of the formation of graphene from graphite. Source: Reprinted with permission from Ref. [108].

Different synthesis strategies have been used in the literature to obtain single- or few-layer graphene formed by several graphene sheets stacked together [105,106,107]. The graphite oxide route is probably the most promising technique for the large-scale production of graphene [108]. Typically, graphite is submitted to an oxidation process that introduces oxygen-containing functional groups on its surface (Figure 7). This process generates a lot of structural changes that disrupt the conjugated electronic structure and lead, consequently, to a significant reduction in electrical conductivity. The oxidized structure can be easily exfoliated to individual graphene oxide (GO) sheets that can be subsequently reduced to graphene via elimination of the oxygen functionalities in order to partially restore the electrical conductivity. On the other hand, graphene and its derivatives often require a modification of their surface chemistry through a functionalization process in order to improve their dispersibility and to enhance interfacial interactions with the surrounding medium [109]. Examples of the surface functionalization of graphene and derivatives have been described in the literature [110,111].

In the field of dentistry, graphene and its derivatives have emerged as one of the most innovative materials on account of their high mechanical stiffness, large surface area and ease of functionalization. The dental applications, including those in dental implants as nanocoatings or antibacterial agents, are described in the work of Li et al. [112].

Several studies highlight the potential of the oxidized graphene derivative, namely graphene oxide (GO), in dentistry due to its biocompatibility and its excellent antibacterial properties. The oxygen-containing functional groups on its surface, in addition to a large surface area, introduce some hydrophilicity, favorable for better adhesion to the titanium surface. Moreover, GO can be functionalized with a wide range of molecules in order to increase its biocompatibility and to help osseointegration [113,114,115,116,117]. A reduced-GO titanium surface has also been shown to accelerate the osseointegration and dental tissue regeneration [118].

The use of GO as nanoscale reinforcing fillers is shown in the work of Li et al. [119], who prepared hydroxyapatite (HA) coatings on a titanium substrate using a cathodic electrophoretic deposition process that ensures a uniform coating with a controlled thickness. The incorporation of GO at 2 and 5 wt.% into HA coatings is seen to reduce the crack formation and increase the coating adhesion strength. The synthesis of HA/GO composites through the sol–gel process, with varying amounts of GO (1% to 3%) and coated on 316L SS via a spin coating technique, was carried out by Sebastin and Uthirapathy [120]. The incorporation of GO into HA increases the hardness of the material that increases linearly with the GO content and decreases the porosity of the resulting composite, as revealed by scanning electron microscopy. The bonding strength of HA and the substrate is seen to increase upon the incorporation of GO, which is attributed to the to the attraction of the calcium ions by the functional groups of the graphene oxide and to their anchorage to the metallic surface. GO and gelatin composite coatings have also been used to modify the surface of titanium implants [121]. The composites exhibit strong antibacterial properties against Staphylococcus aureus and show an obvious inflammatory reduction.

In recent years, the focus of innovative research has been on graphene quantum dots (GQDs) that belong to the new form of carbon nanomaterials called carbon dots, which are zero-dimensional structures with an exceptionally small size (a few nanometers) and intrinsic photoluminescence properties.

CDs can be obtained by breaking different carbon resources, like carbon, graphite, carbon nanotubes, fullerenes, graphene oxide, etc., using arc discharge, laser ablation or electrochemical oxidation (top-down approach). The second synthetic route (bottom-up approach) commonly uses, as starting materials, carbohydrates such as glucose, lactose and maltose or biopolymers based on carbohydrates such as cellulose or chitosan. In the bottom-up strategy, CDs are obtained by dehydration and further carbonization [122,123,124,125].

**Figure 8:**
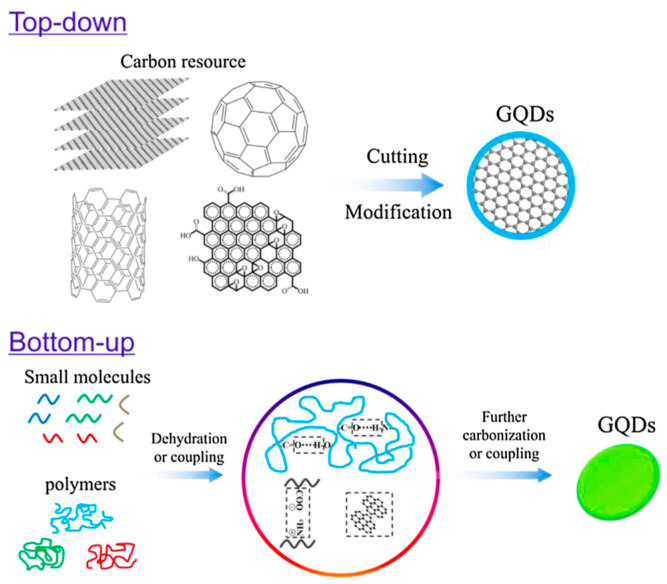
Main synthesis routes of graphene quantum dots. Source: Reprinted with permission from Ref. [124].

The two main approaches for the synthesis of graphene quantum dots (GQDs) are illustrated in Figure 8. Their potential in different fields such as energy, optical and medical fields has been highlighted in several reports [125,126,127,128]. In medical research, the applications of CDs including GQDs, such as bioimaging, biosensing, tissue engineering, drug delivery or antibacterial treatment, have been widely mentioned in the literature [128,129,130,131,132,133,134,135,136].

One critical challenge in the field of dentistry is to treat implant-related bacterial infections arising from biofilms that can enhance the bacterial resistance by inhibiting antibiotic action and cause dental implant failure [137]. Carbon quantum dots and especially graphene quantum dots, which have been shown to present great potential as antibacterial and anti-biofilm agents [138,139,140], will find a wide applicability in reducing the adhesion of biofilms on dental surfaces.

Owing to their mechanical strength, their sheet structure, their ability to be functionalized, their biocompatibility as well as their antibacterial and antibiofilm properties that are essential for promoting the osseointegration process, graphene-based nanomaterials are obviously attracting growing interest in many dental fields and especially in the area of titanium dental implants (Table 1).

## 4. Potential Toxicity of the Implant Materials and Nanoparticle Coatings

Dental implants and nanotechnology have revolutionized the world of dentistry. However, with their growing use comes concern about their possible long-term health effects.

Regarding titanium and its alloys, the release of metal particles from their surface to the oral environment has been demonstrated, and several reviews have analyzed the current literature in order to identify the possible causes of Ti particle formation from dental implants [141,142,143,144,145,146,147]. It is suggested that the titanium oxide layer present on the implant surface may be dissolved by the saliva and bacteria, thus initiating a corrosion process. It is also mentioned that implant insertion into the bone causes oxide-layer breakdown and material removal from the implant surface [141]. It is also made clear that the peri-implantitis sites caused by an inflammatory process exhibit a higher number of particles compared to healthy implants [142] and that the accumulation of titanium particles in surrounding tissues is related to the corrosion of the metal structure. Moreover, it is concluded that the shedding of titanium particles likely starts at the time of implant placement and continues under forces of mastication [145]. The released Ti particles that are essentially of micrometric size are not confined to the tissues surrounding the implant but can migrate through the blood to different organs. The smaller particles are expected to produce greater toxicity [147], and their size decreases with an increase in distance from the implant [146]. Measurements carried out by Shelli et al. [148] on humans bearing orthopedic titanium implants reveal high titanium levels in the tissue surrounding the implant and a positive correlation between titanium levels and time of implant in the body. This would indicate a constant release of titanium particles, which may cause long-term pathologic consequences. Additionally, the authors describe the case of titanium toxicity in a patient who presented a dysfunction in the central nervous system after the implantation of a titanium-based carotid stent.

The potential neurotoxicity of titanium dioxide nanoparticles has also been reviewed [149,150], but according to the authors, further standardized investigations are required because the conclusions from various studies are somewhat conflicting, resulting from the use of different experimental parameters. An interesting study by Dhein et al. [151] compares the cyto- and genotoxicity of titania and zirconia particles as well as the bacterial adhesion on dental titanium and zirconia implants. Since the particle size is expected to influence the toxicity of particles, their study was carried out using periodontal ligament cells exposed to micro- and nanoparticles. The results indicate that the TiO_2_ nanoparticles induced about six-times higher cytotoxicity than the TiO_2_ microparticles (the EC_50_ values are, respectively, 15 and 92 mg/mL); both oxidized Ti particles are, nevertheless, less cytotoxic than the elementary Ti nanoparticles (the EC_50_ value is 3 mg/mL). Interestingly, the bacterial adhesion to ZrO_2_ implants is significantly reduced with regard to Ti implants, but this result has to be checked in a clinical context.

The structural parameters of nanoparticles affecting their toxicity for biomedical applications were highlighted in a recent review [152]. The size and the surface area of the particles are crucial parameters with regard to the toxicity aspects. Small particles can infiltrate the body tissues and their surface area that strongly increases with their reduced size, allowing a larger interface between the nanoparticles and the biological system. The aspect ratio of the nanoparticles is also an additional parameter that can affect their toxicity. It is the case that anisotropic particles like carbon nanotubes or graphitic structures can have a large contact area with tissues and cells due to their high aspect ratio. While the high aspect ratio of anisotropic particles has a strong positive impact on the mechanical and electrical performances of composite materials, it could have adverse health effects on account of the large interactive surface with the living components, making anisotropic particles more toxic than the spherical nanostructures. Moreover, carbon nanotubes (CNTs) have become a major concern due to their structural similarity with asbestos. An interesting comparison between the two species that highlights similarities and chemical differences was carried out by Fubini et al. [153]. The two materials share an elongated fibrous form, but asbestos is very hydrophilic, while CNTs are highly hydrophobic unless oxidized or functionalized. The authors also stress the role of iron in asbestos toxicity and mention the presence of iron in CNTs as a residue of the catalyst used in their synthesis. In addition to the presence of metal, the toxic responses of CNTs have been shown to be influenced by other factors such as structural defects in the graphene framework, the tube length or the surface-oxygenated functionalities. In the work of Fenoglio et al. [154], aiming to understand the physicochemical characteristics of CNTs that determine their toxicity, it appears that the structural defects may be one of the major factors that impact their toxic potential. Safety concerns about the risk of exposure to graphene-based materials have also been raised on account of the growing interest in these nanoparticles in biomedical applications [155]. It is reported in several studies that many factors can interact with the biological systems, namely the size of the particles, their concentration, state of dispersion, surface functionalization as well as their shape, which includes the lateral dimensions and the presence of sharp edges able to penetrate the cell membranes [156,157,158,159,160]. The recent work of Kharlamova and Kramberger [161] states that some issues of cytotoxicity can be solved through chemical functionalization carried out by covalence, noncovalence, intercalation, substitution or filling. The proper surface functionalization of carbon-based nanomaterials is also required for the innovative upgrading of dental implants [162]. Nevertheless, from all reports, it seems clear that an understanding of the correlation between the nanoparticle characteristics and their related cytotoxicity is absolutely needed before clinical use.

## 5. Conclusions

The surface modification of dental implants is required to improve bone–implant adhesion and avoid bacterial infiltration. Nanoscale modifications with the use of nanoparticles have attracted a great deal of interest since they can offer a high surface area and adequate interfaces due to their ability to be functionalized with various chemical groups. Moreover the potential of some nanomaterials as antibacterial and drug delivery agents opens the way for preventing bacterial infection and implant loss. Nanoparticles applied as coatings have been shown to improve the corrosion resistance of the substrate surface and to enhance the osseointegration process.

This paper reviewed various nanoscale surface modifications performed in titanium dental implants. Recent nano-engineering techniques were presented and discussed through results presented in the literature. These techniques include the generation of nanosized spherical particles by the sol–gel process, the formation of titanium dioxide nanotubes by electrochemical anodization and the use of carbon nanomaterials such as carbon nanotubes or graphene and its derivatives, particularly suited as reinforcing agents of composite coatings owing to their outstanding mechanical properties.

A titanium dioxide coating formed by the sol–gel process or by anodic oxidation, being compact and homogeneous, increases the corrosion resistance and biocompatibility and prevents the release of titanium particles from implants. This has renewed the interest in hydroxyapatite (HA), well known to have a limited use as a coating for dental implants due to its low strength and toughness. A TiO_2_ layer inserted between titanium and hydroxyapatite increases the adhesion strength with regard to the HA coating alone. Hydroxyapatite-based nanocomposite coatings based on a combination of HA with nanoparticles (TiO_2_, SiO_2_, carbon-based materials) able to enhance the mechanical properties of HA are expected to have bright prospects in the future.

Among all the nanoparticles, graphene-based nanomaterials and especially graphene oxide (GO) have probably generated the greatest interest in the field of dentistry. GO can be functionalized with various chemical groups, allowing for enhanced solubility, biocompatibility and antimicrobial properties. But the graphene oxide properties, including the antibacterial activity and cytotoxicity, have been observed to depend strongly on several factors, such as the concentration, size, shape and functionalization. The toxicity can be reduced by incorporating the graphenic material in a polymer to form a polymer nanocomposite. Another interesting approach, already applied in polymer nanocomposites, is the use of mixtures of nanoparticles of different morphologies that may exhibit better properties than each single type of particles on account of possible synergistic effects. This will allow for a decrease in the amount of nanomaterial with potential toxicity.

## Figures and Tables

**Table 1:** Summarizes the main properties of common nanoparticles used in dental implants.

Nanoparticles	Coating Technique	Properties	References
TiO_2_	Sol-gel technique	-Produces homogeneous fine-grained titania coatings with a high surface area and almost absence of defects and fissures. -Improves the corrosion resistance of Ti. -Can be used as a buffer layer between the substrate and another coating.	[49,50,51,52]
TiO_2_	Electrochemical anodization	-Allows the growth of 10 nm to 40 μm of compact TiO2 oxide layers. -The presence of fluoride ions allows the formation of tubular structures with, a high surface area, a high structural order and a high biocompatibility. -The nanotubes can be used as drug delivery systems.	[10,38,54,56,60]
Hydroxyapatite (HA)	Sol-gel technique	-Produces uniform and homogeneous coatings however poor adhesion to the substrate and low mechanical properties. -Optimizing the sol-gel processing parameters is required to achieve ideal HA coatings. -HA coated over a TiO_2_ layer on the Ti substrate improves the bonding to the Ti substrate as a result of the affinity of the titania layer toward HA and Ti. -HA-based nanocomposites often display better adhesion and improved mechanical properties than HA coating alone.	[82,85,86,87] [74,88]
Carbon nanotubes	Dip-coating	-Used as reinforcing agents on account of their exceptional mechanical and electrical properties. -The addition of CNTs in HA composite coatings improves the microhardness and elastic modulus. But they have to be used at low content (below 1 wt%) to avoid the formation of agglomerates. -Surface functionalization of CNTs can provide the desired surface chemistry in order to improve their dispersion and to allow their use at higher contents for enhanced mechanical properties.	[95,96,97] [101] [92,93,96,98]
Graphene-based materials	Electrophoretic deposition Sol-gel process	-Most promising materials on account of their outstanding mechanical, electrical and thermal properties. -But GO, is more suitable for dental applications since it brings hydrophilicity required to improve adhesion to the substrate. -GO-functionalization via its reactive oxygen groups increases its biocompatibility, its antibacterial properties and a better integration with the surrounding bone tissues. - Compared with pure HA coating GO/HA composite coatings display enhanced adhesion strength and corrosion resistance and reduce coating cracks. -Incorporation of GO into HA increases the hardness of the material and the corrosion resistance with a superior bonding strength between HA and the substrate. -Graphene quantum dots which are the new comers in the graphene family offer great promise as anti-biofilms and anti-bacterial agents.	[106,107] [114] [119] [120] [138,139,140]

## Data Availability

Data is contained within the article.
